# Laguerre Filter Analysis with Partial Least Square Regression Reveals a Priming Effect of ERK and CREB on c-FOS Induction

**DOI:** 10.1371/journal.pone.0160548

**Published:** 2016-08-11

**Authors:** Takamasa Kudo, Shinsuke Uda, Takaho Tsuchiya, Takumi Wada, Yasuaki Karasawa, Masashi Fujii, Takeshi H. Saito, Shinya Kuroda

**Affiliations:** 1 Department of Biological Sciences, Graduate School of Science, University of Tokyo, Hongo 7-3-1, Bunkyo-ku, Tokyo, 113-0033, Japan; 2 Department of Computational Biology, Graduate School of Frontier Sciences, University of Tokyo, Hongo 7-3-1, Bunkyo-ku, Tokyo, 113-0033, Japan; 3 Department of Neurosurgery, Faculty of Medicine, University of Tokyo, Hongo 7-3-1, Bunkyo-ku, Tokyo, 113-0033, Japan; 4 CREST, Japan Science and Technology Corporation, Bunkyo-ku, Tokyo, 113-0033, Japan; 5 Division of Integrated Omics, Research Center for Transomics Medicine, Medical Institute of Bioregulation, Kyushu University 3-1-1 Maidashi, Higashi-ku, Fukuoka, 812-8582, Japan; University of the Basque Country, SPAIN

## Abstract

Signaling networks are made up of limited numbers of molecules and yet can code information that controls different cellular states through temporal patterns and a combination of signaling molecules. In this study, we used a data-driven modeling approach, the Laguerre filter with partial least square regression, to describe how temporal and combinatorial patterns of signaling molecules are decoded by their downstream targets. The Laguerre filter is a time series model used to represent a nonlinear system based on Volterra series expansion. Furthermore, with this approach, each component of the Volterra series expansion is expanded by Laguerre basis functions. We combined two approaches, application of a Laguerre filter and partial least squares (PLS) regression, and applied the combined approach to analysis of a signal transduction network. We applied the Laguerre filter with PLS regression to identify input and output (IO) relationships between MAP kinases and the products of immediate early genes (IEGs). We found that Laguerre filter with PLS regression performs better than Laguerre filter with ordinary regression for the reproduction of a time series of IEGs. Analysis of the nonlinear characteristics extracted using the Laguerre filter revealed a priming effect of ERK and CREB on c-FOS induction. Specifically, we found that the effects of a first pulse of ERK enhance the subsequent effects on c-FOS induction of treatment with a second pulse of ERK, a finding consistent with prior molecular biological knowledge. The variable importance of projections and output loadings in PLS regression predicted the upstream dependency of each IEG. Thus, a Laguerre filter with partial least square regression approach appears to be a powerful method to find the processing mechanism of temporal patterns and combination of signaling molecules by their downstream gene expression.

## Introduction

Signal transduction networks have a “bow-tie” architecture. That is, a relatively small set of molecules comprising the signal network are capable of both receiving information from diverse environmental sources and inducing a wide variety of gene expression patterns [1]. This raises questions as to how signals are selectively encoded by a limited number of signaling molecules and selectively decoded by their downstream targets. The recent studies have shown that different combinations of signaling molecules, as well as the time series of signaling, play important roles in transmission of complex information [2–7]. Part of understanding a complex system is to decode the relationship between inputs and outputs (IO relationship). Dynamic modeling is one approach that can be applied to effectively identify IO relationships. In this study, we focus on using data-driven dynamics to look at IO relationships between MAP kinases (MAPKs) and CREB (inputs) and the immediate early gene products (IEGs; outputs), in PC12 cells. We chose to focus on the signaling system that processes the information on cell fate decisions in a number of molecular species.

A number of methods for dynamic modeling have been proposed but each method has advantages and disadvantages. Kinetic modeling based on biochemical reactions from the literature is a typical modeling approach adopted in systems biology and can perform well when sufficient knowledge of a signaling network is available [8, 9]. However, kinetic modeling is not applicable if knowledge of the signaling pathway is insufficient. By contrast, since data-driven models infer and identify IO relationships by learning from the data sets, they can be suitable even in cases where knowledge of a signaling pathway is not adequate [10]. Nevertheless, there remains some problems with data-driven modeling. Although there is often significant nonlinearity in biological reactions, identifying nonlinear IO relationships using data-driven modeling is not easy as compared to identifying linear IO relationships due to its tendency for having a large model space to explore. In addition, nonlinear data-driven modeling requires quantitative measurement of signaling activities over time, and under various conditions or following various perturbations. Thus, nonlinear data-driven modeling based on experimental data remains a big challenge.

With all this in mind, we sought to develop a nonlinear data-driven modeling approach that can help address how changes in the time series of signaling, as well as the combination of multiple signaling inputs, are decoded by downstream targets. We previously used a nonlinear autoregressive exogenous model (hereafter, “nonlinear ARX model”) to analyze the same signaling network explored in this study [11]. The nonlinear ARX model we developed consists of a combination of the linear ARX model itself and nonlinear transformation of the input using the Hill equation. Our nonlinear ARX model was able to reproduce the time series of immediate early genes (IEGs) in response to signals. However, with this approach, interpretation of the response function is difficult, as nonlinearity of the ARX is heuristically introduced by nonlinear transformation of the input. Another complication of the approach is that the nonlinear ARX model includes autoregressive components. In other words, the model reproduces the time series of each IEG’s response of future time not only based on the inputs pMAPKs and pCREB of past time, but also based on the IEGs themselves of past time. To avoid such issues, we would like to establish a more generalized approach for nonlinear-data driven modeling framework by which parameters can be trained with a limited experimental data.

In this study, we used a Laguerre filter [12] with partial least square (PLS) regression to infer nonlinear IO relationships from time series data. A Laguerre filter is a time series model that includes nonlinear IO relationships. The Laguerre filter is yielded by the Volterra expansion of nonlinear system and consists of linear and nonlinear components, which are expanded by Laguerre basis functions and correspond to the response functions of linear and nonlinear system. The expansion coefficients of the Laguerre basis functions are determined using a PLS regression method [13]. We applied the Laguerre filter approach to identify the IO relationships among MAP kinases, CREB and IEGs, and found a priming effect of phosphorylated ERK (pERK) and phosphorylated CREB (pCREB) on c-FOS induction from the second-order Volterra kernel.

## Results

### Representation of a nonlinear system using a Laguerre filter approach

**Fig 1:**
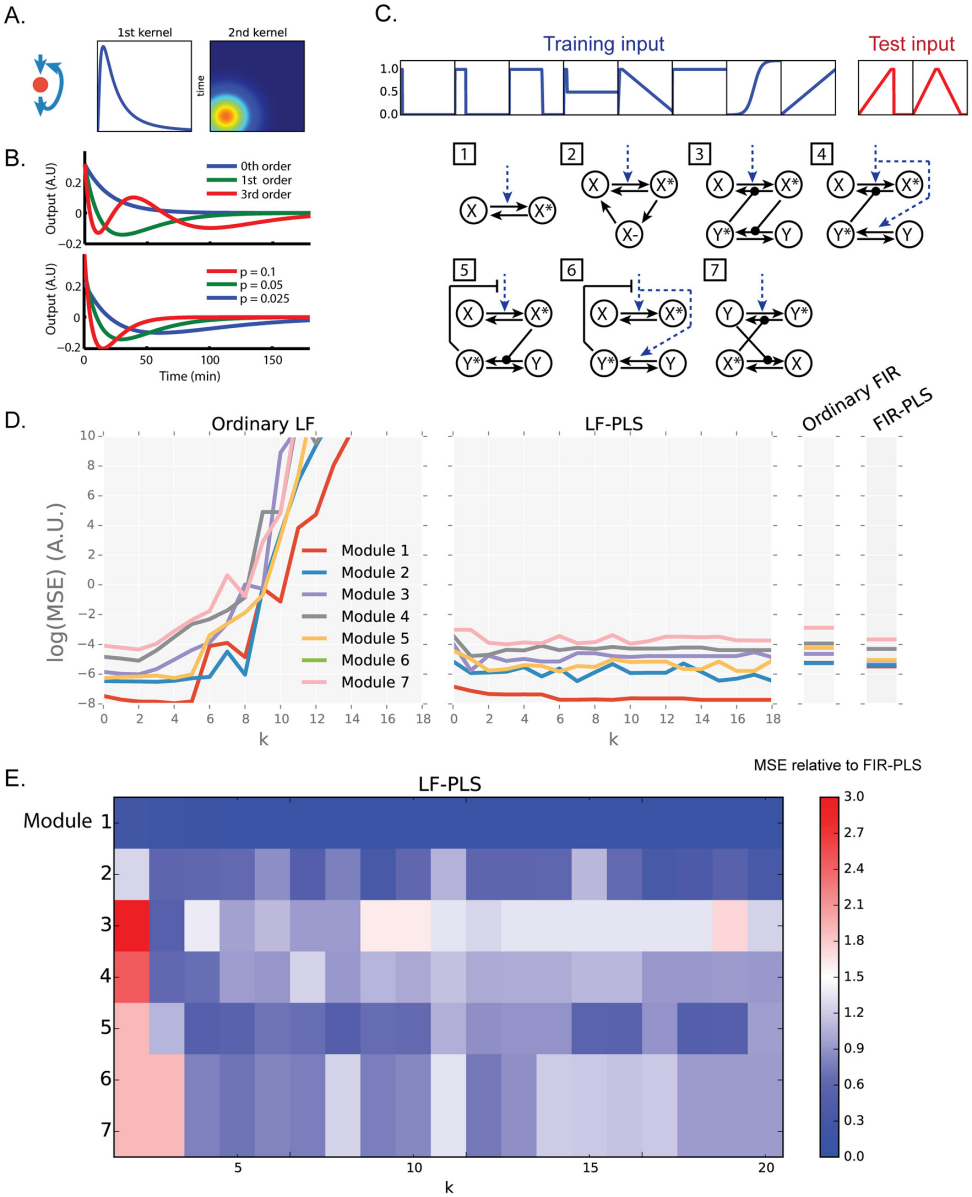
Development of Laguerre filter with PLS regression. (A) A schematic representation of 1^st^ and 2^nd^ Volterra kernels. If a nonlinearity exists in a system (such as the negative feedback as illustrated), the non-zero values appears in 2^nd^ kernel. (B) Examples of Laguerre polynomial functions with different parameters. (C) The network modules and input patterns used to create artificial input-output time series. We used 10 input patterns in total, including 2 input patterns for test models. 7 modules represent some of the commonly found biochemical network architectures. (D) The log-scale mean square error (MSE) of the four models for each modules. (E) Heatmap showing MSE of LF-PLS normalized by MSE of FIR-PLS.

A Volterra model is a canonical representation of a nonlinear system. We constructed a model based on a second-order Volterra model

$$y(t)\approx \int d\tau_{1}k_{1}(\tau_{1})u(t-\tau_{1})+\int d\tau_{1}d\tau_{2}k_{2}(\tau_{1},\tau_{2})u(t-\tau_{1})u(t-\tau_{2}),$$ (1)

where *u*(*t*) and *y*(*t*) are an input and output, respectively. For simplicity, we set the constant term to 0 without loss of generality. The above equation consists of the sum of single and double convolution integrals, where *k*_1_ and *k*_2_ are the Volterra kernels. The first-order term, a single convolution integral, corresponds to the IO relationship of linear time invariant systems, and represents a linear component of the Volterra model. The second-order term, a double convolution integral, represents a nonlinear component of the Volterra model. The second-order Volterra kernel *k*_*2*_ represents the nonlinear way in which two inputs of pulse affect an output (Fig 1A). The 2nd order Volterra kernel *k*_2_ is interpreted as the interaction of two impulses, which can be represented by 2 dimensional symmetric functions about the diagonal. In this study, interactions between different inputs are ignored for simplicity. Thus, the interaction of two impulses with lag *τ* of the same input is represented by a line through point *τ* of the x- or y-axis, in parallel with the diagonal. For example, the response of two impulses with lag *τ* can be represented by *k*_1_(*t*)+*k*_1_(*t*−*τ*)+*k*_2_(*t*,*t*)+2*k*_2_(*t*,*t*−*τ*)+*k*_2_(*t−τ*,*t*−*τ*).

The Volterra kernels are determined by truncating the lower order series of the Laguerre basis expansion. The *n*-th order Laguerre polynomial function is,

$$l_{n}\left(t\right)=\sqrt{2p}e^{pt}\frac{1}{\left(n-1\right)!}{\left(\frac{d}{dt}\right)}^{n-1}t^{n-1}e^{-2pt},$$ (2)

where the time-scale of exponential decline is controlled by parameter *p*. The complexity of dynamics is dependent on *n*-th order of the function (Fig 1B). This function was chosen as a basis function due to its relaxing dynamics, a property that is appropriate to express the dynamics of many physiological systems [14]. The Laguerre functions up to *k*-th order were used as the basis functions; the *n*-th order Laguerre basis functions defined in [0,∞] are orthogonal to each other, and each parameters can thus be estimated independently. The Volterra model, combined with Laguerre basis expansion of kernels, comprises the filter itself. The expansion coefficients of the Laguerre basis functions are estimated by regression.

To assess the applicability of the model for signaling study, we applied this framework to a number of datasets computationally produced by modeling the network architectures commonly found in signaling networks with a simple set of input dynamics adapted from previous work (Fig 1C) [15]. We used seven network modules which include one- or two-component regulatory modules with various regulatory mechanisms, thus expected to cover different types of nonlinearity often found in signaling networks. For each modules, the output X* is simulated by feeding the input time-series which comprises of 10 different input patterns with 301 data points each. The model is trained with eight input patterns and tested against two input patterns.

As we increased *k*, the number of Laguerre basis functions used for expansion, the mean square error (MSE) for test inputs of ordinary Laguerre filter (Ordinary LF) increased drastically (Fig 1D). This implies the difficulty of parameter estimation of ordinary LF under a limited amount of training data in cell signaling systems. We thus applied partial least square (PLS) regression to estimate the expansion coefficients, hereafter called LF-PLS model, which successfully suppressed the over-fitting of the model even with large *k*. We also adopted the linear models for comparison, a finite impulse response model with ordinary regression (Ordinary FIR) and a finite impulse response model combined with PLS regression (FIR-PLS). LF-PLS and the ordinary LF can describe the nonlinear IO relationships based on extension of the impulse response via the Volterra expansion, whereas the ordinary FIR and FIR-PLS describe only linear IO relationships based on impulse response. Similarly to the nonlinear LF, but to a lesser extent, the use of PLS regression reduced the MSE for test inputs for these linear models (Fig 1D). This result suggests the applicability of the PLS regression in reducing over-fitting of the parameter estimation particularly for the nonlinear modeling. We further compared the MSE for test inputs of LF-PLS to those of FIR-PLS (Fig 1E). In all of the modules, the predictability of LF-PLS is comparable or better than those of FIR-PLS, except for the models with low *k*. Again, the PLS regression suppresses the over-fitting of the models, and thus MSE for test inputs seems to converge for the models with *k* bigger than 4, although there are some local minima probably caused by initial value problem. Given that, hereafter we set *k* to 10 and trained models several times with randomized initial values in order to achieve a robust parameter estimation.

### The MAPK, CREB, IEG signaling network

**Fig 2:**
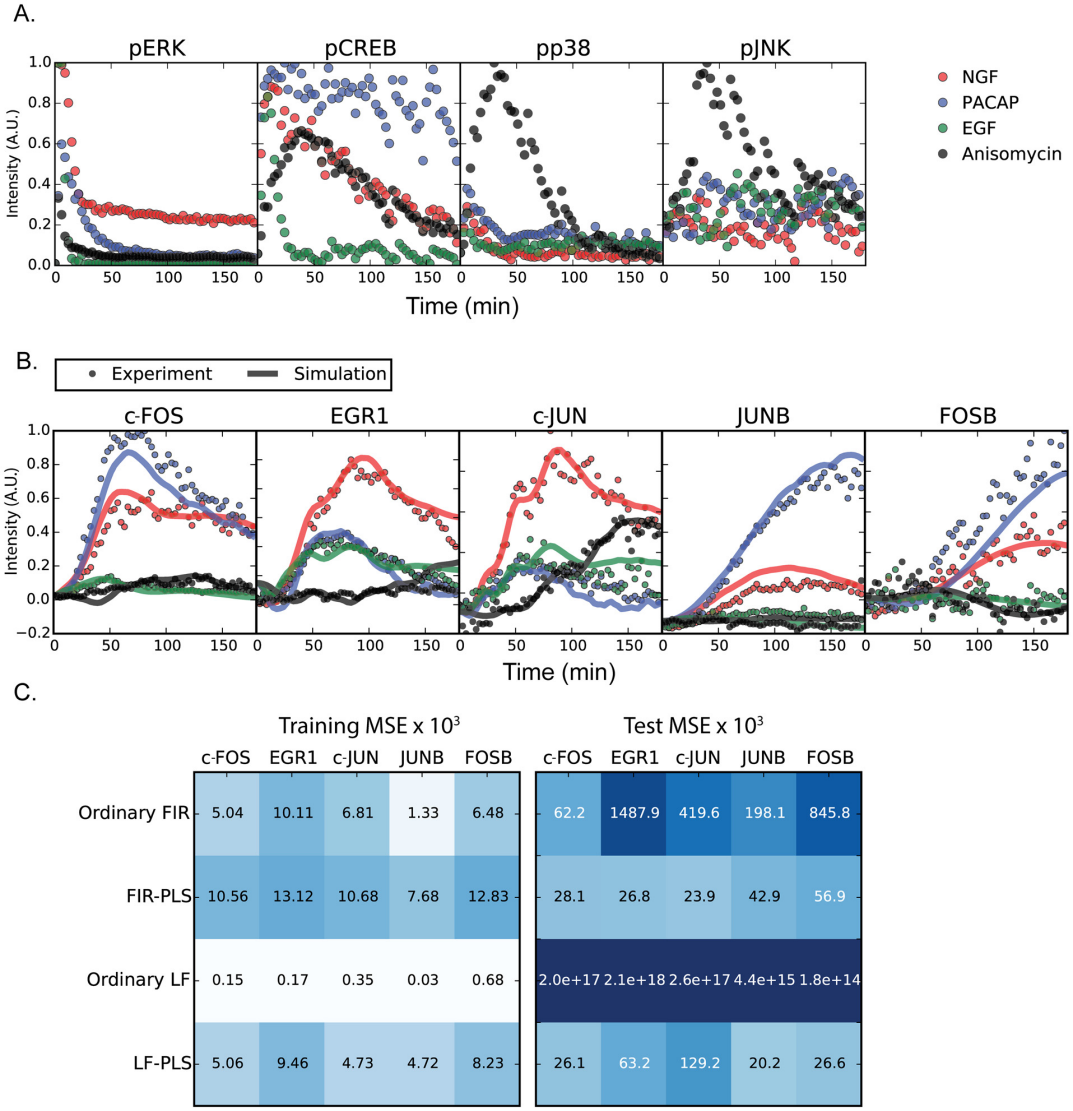
pMAPKs and pCREB as inputs and immediate early gene expression as outputs. (A) The time series of pMAPKs and pCREB in response to NGF (5 ng/ml, red), PACAP (100 nM, blue), EGF (5 ng/ml, green), and anisomycin (50 ng/ml, black) were measured by quantitative image cytometry (QIC) at 3-min intervals for 180 min. One time series with respect to each growth factor is shown to facilitate visualization. Other time series are shown in S1 Fig. (B) The time series of the immediate early genes (IEGs) measured by QIC (circles) are shown, together with simulated results of Laguerre filter combined with partial least square (PLS) regression (LF-PLS) (solid lines). The color codes are same as described for panel A. These data, together with responses to other doses of the growth factors (S1 Fig), were used for parameter estimation of LF-PLS. The circle plots in panels (A) and (B) were created based on data from [11]. (C) The MSE of leave one out error of cross validation (LOO CV) for each method. The full MSE of LOO CV of Laguerre filter is shown in S6 Fig. The numerical value of MSE of training data set and LOO CV are shown in S1 and S2 Tables, respectively.

Growth factors induce phosphorylation of signal transduction molecules. Subsequently, these molecules lead to increased levels of IEG proteins, presumably via induction of transcription. We used quantitative image cytometry (QIC) [16], which integrates a quantitative immunostaining technique and a high-precision image-processing algorithm for cell identification, to measure a set molecules over time. Specifically, we measured levels of the phosphorylated forms of four signaling molecules, phosphorylated ERK (pERK), JNK (pJNK), p38 (pp38) and CREB (pCREB), and protein levels of five IEGs, which are c-FOS, EGR1, c-JUN, JUNB and FOSB (Fig 2A and 2B and S1 Fig). Hereafter, we use “pMAPKs” to refer collective to three MAP kinases, pERK, pJNK and pp38, and “IEGs” to refer to c-FOS, EGR1, c-JUN, JUNB and FOSB. The data set used to train the model is identical to the data set used in our previous work [11]. The molecules were measured every 3 min up to 177 min in 7 conditions, which are NGF (5 ng/ml, 0.5 ng/ml), PACAP (100 nM, 1 nM), EGF (5 ng/ml, 0.5 ng/m), and anisomycin (50 ng/ml).

### Performance of the Laguerre filter combined with PLS regression

We applied the Laguerre filter in combination with PLS regression (LF-PLS) and set each of five IEGs as outputs and the four molecules, pMAPKs and pCREB, as inputs (Fig 2A). The LF-PLS successfully reproduced the time series of IEGs (Fig 2B and S1 Fig). To assess the efficacy of this framework, the LF-PLS model was compared with the other models. To evaluate performance among the four models, we calculated the MSE of leave-one-out cross-validation (LOO CV) (Fig 2C). For each round of LOO CV, one out of 7 samples, corresponding to treatment with 4 different growth factors (see Materials and Methods), is chosen as the LOO CV test. The residual distributions for each LOO CV and estimated parameters of LOO CV and final model are plotted (S2 and S3 Figs). The MSE of the LOO CV test of LF-PLS was smaller than that of ordinary LF (Fig 2C). Similarly, the MSE of the LOO CV of FIR-PLS was smaller than that of ordinary FIR. This indicates the efficacy of the PLS regression compared to the ordinary regression.

The MSEs of LOO CV of the LF-PLS for EGR1 and c-JUN were larger than those obtained with the FIR-PLS. This suggests that the nonlinearity of EGR1 and c-JUN is relatively weak by which can be expressed by linear models or the nonlinear models were not trained accurately with a given training dataset. The ordinary LF and LF-PLS are nonlinear models that include a linear model and able to reproduce an IO relationship of the linear model. However, a nonlinear model needs to estimate a larger number of parameters than a linear model from the finite size data set. Thus, the accuracy of parameter estimation of a nonlinear model can sometimes be lower than a linear model, when small-size data set is used. This indicates that when the IO relationship is linear, the results obtained using LF are not always better than those obtained using linear models. However, for c-FOS, JUNB and FOSB, the predictability of LF-PLS was better than that of FIR-PLS (Fig 2C and S4 Fig). The Mann—Whitney U-test was used to examine a significant difference of each median of squared residuals, which were obtained from LOO CV, between LF-PLS and FIR-PLS. The significant differences appeared in c-FOS, EGR1, c-JUN, and FOSB in significant level 0.05, and JUNB in significant level 0.1 (S3 Table). This result indicates that, even with a limited amount of training data that we can obtain from the experiment, our framework using nonlinear LF-PLS can be more appropriate than using linear models for describing the IO relationship between some of the signaling pathways and their transcriptional output.

The residual distributions indicate (S2 Fig) that the underestimation and overestimation in LF-PLS of c-FOS, JUNB and FOSB less frequently occurred and were smaller than those in FIR-PLS. In FIR-PLS of EGR1 and c-JUN, the relationships is upside down. A few of coefficient parameters estimated by PLS regression of final model correspond to outliers for the distributions of coefficient parameters, which was estimated by PLS regression in each LOO CV test (S3 Fig). The number of corresponding outliers are 8, 11, 7, 0 and 0 for c-FOS, EGR1, c-JUN, JUNB and FOSB, respectively.

### Interpretation of the 1st and 2nd order Volterra kernels

**Fig 3:**
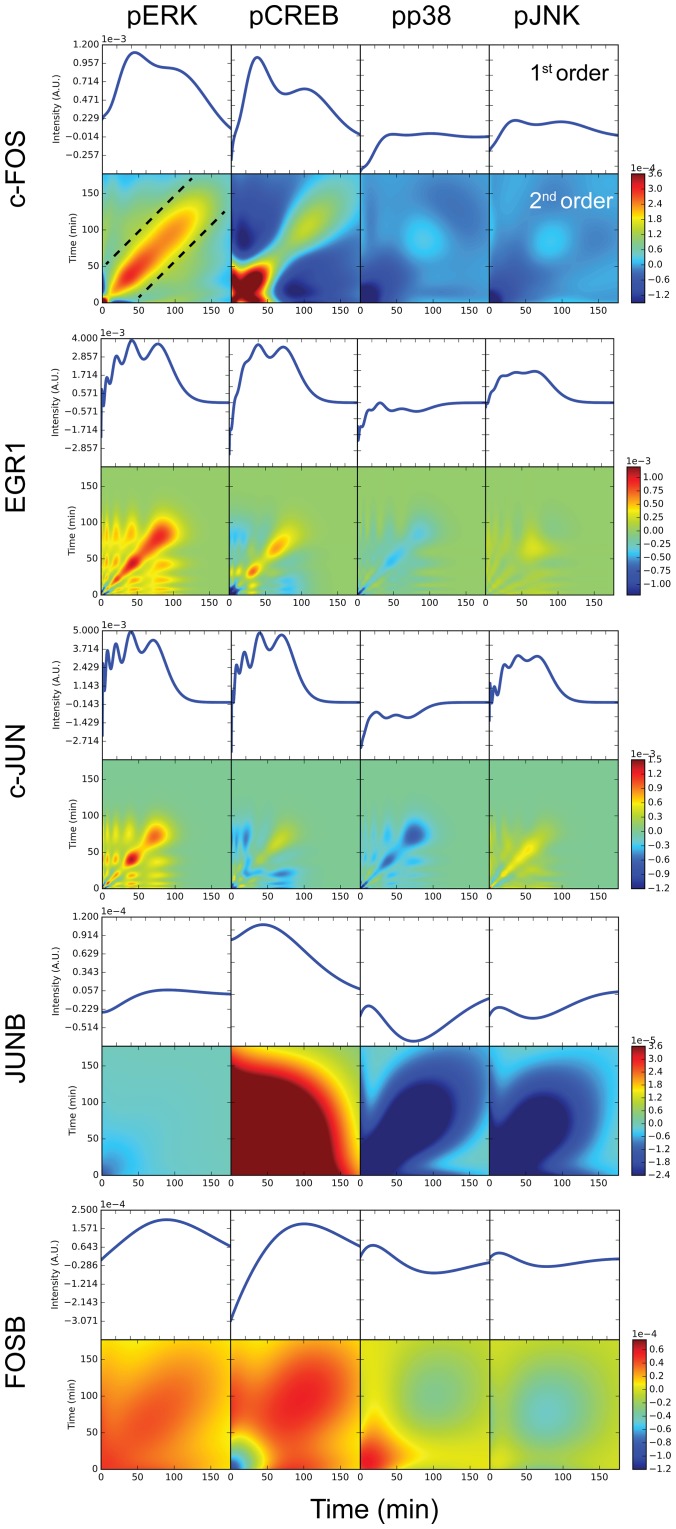
First and second order Volterra kernels. The solid line in upper panels indicates the 1st order Volterra kernel *k*_1_ in the time space. The color in the lower panels indicates the 2nd order Volterra kernel *k*_2_ in the time space.

The 1st and 2nd order Volterra kernels *k*_1_, *k*_2_ of each output are shown in Fig 3. The 1st order Volterra kernel *k*_1_ is equivalent to the impulse response function to the input of just one impulse whose effect is linear. The 2nd order Volterra kernel *k*_2_ represents the nonlinear effect of the input of two impulses. In response to the impulsive input of pERK, the peak times of 1st order Volterra kernel *k*_1_ of c-FOS, EGR1 and c-JUN were earlier than those of JUNB and FOSB. This result indicates that c-FOS, EGR1 and c-JUN respond to pERK quicker than JUNB and FOSB. The 1st order Volterra kernel *k*_1_ of EGR1 and c-JUN decayed quickly, whereas the 1st order Volterra kernel *k*_1_ of c-FOS and FOSB decayed slowly. Thus, c-FOS responds to pERK more quickly and sustained manner. The initial rise of FOSB was slower than those of c-FOS, EGR1 and c-JUN, and the 1st order Volterra kernel *k*_1_ of JUNB was weaker than those of c-FOS, EGR1 and c-JUN. The different characteristics of FOSB and JUNB as compared with c-FOS, EGR1 and c-JUN might reflect indirect regulation of FOSB and JUNB by pMAPKs. Indeed, unlike c-Jun, which is activated by JNK, JUNB is not directly induced by JNK [17]. In addition, a putative DEF (docking for ERK FXFP) domain is present in c-FOS but not in FOSB [18].

The 2nd order Volterra kernel *k*_2_ of each output is almost everywhere positive, indicating the IO property of double impulse inputs in which the first impulse enhances second lagged impulse. This indicates a positive synergistic effect of double impulse inputs, a phenomenon referred to hereafter as the priming effect. The intensity of the 2nd order Volterra kernel *k*_2_ becomes lower with increasing distance from the diagonal, indicating that the nonlinear effect becomes smaller as the lag between the first and second impulse increases.

In response to the pCREB input, the 1st order Volterra kernel *k*_1_ of each output behaved similarly as compared with the response with pERK as the input, with the exception of the response of JUNB. In the off-diagonal or around origin of 2nd order Volterra kernel *k*_2_ of each output, again with the exception of JUNB, the 2nd order Volterra kernel *k*_2_ was negative. This indicates that the IO property of double impulse inputs, in which the first impulse reduces the effect induced by a second, lagging impulse, highlighting that regulation of IEGs by pERK is distinct from regulation by pCREB.

In response to each of pERK and pCREB inputs, an intricate, multimodal pattern appeared in the 1st and 2nd order Volterra kernels *k*_1_, *k*_2_ of EGR1 and c-JUN. This may suggest the over-fitting in the estimation of EGR1 and c-JUN.

**Fig 4:**
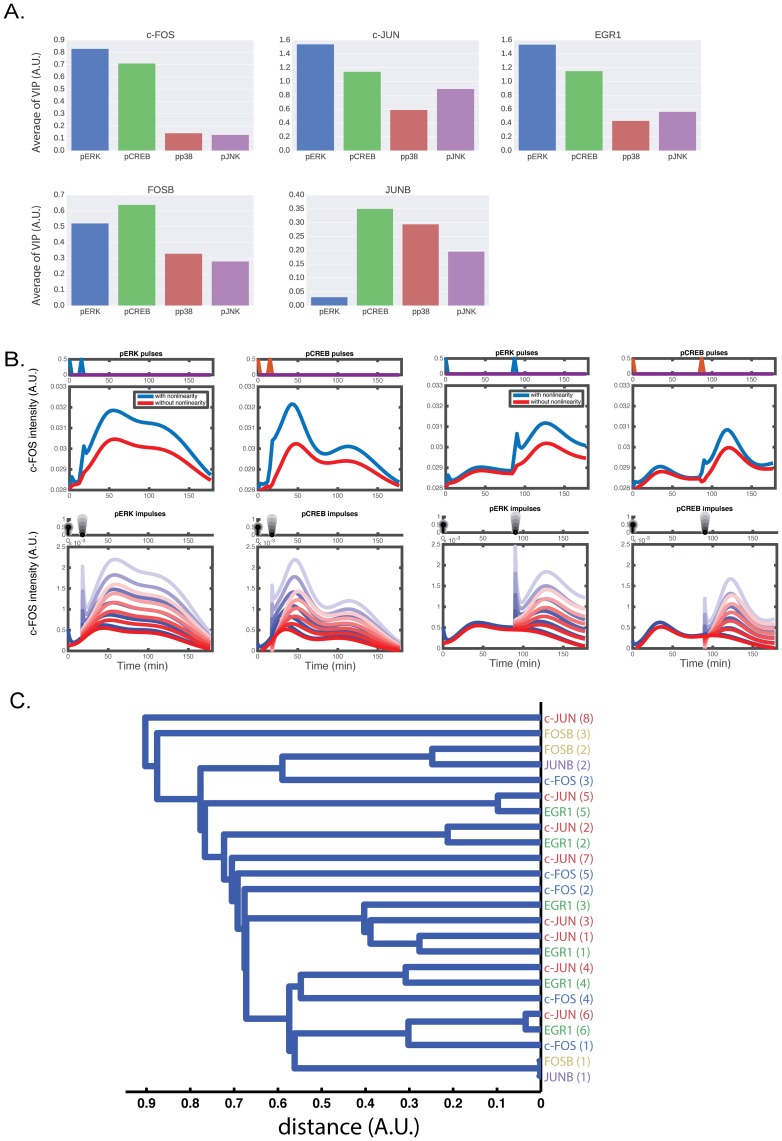
The mean VIP score and a dendrogram based on hierarchical clustering of input loading. (A) Averaged VIP scores of the input. (B) Computational and theoretical c-FOS time course given two pulsatile and impulsive pERK and pCREB inputs. For the top panels, c-FOS time course was simulated when two pulses were given as inputs. The bottom panels show the theoretical impulse responses when two impulses were given as inputs. The amplitude of the second impulse was tuned from 0 to 1. The calculation with nonlinear model is shown in blue and without nonlinearity is shown in red. (C) Hierarchical clustering as applied to the input loadings. The distance between two samples is defined as 1-*r* where *r* is the Pearson correlation. The distance between two clusters is defined as the shortest distance between two samples drawn from each of the clusters. The leaf nodes represent the output loadings, and the order of principle components is described in parenthesis. The loadings and scores of the input and output used for principal component analysis are shown in S5 Fig.

In response to the pp38 or pJNK inputs, the intensities of the 1st order Volterra kernel *k*_1_ and the 2nd order Volterra kernels *k*_2_ were lower as compared to what is observed for pERK and pCREB. Thus, the role of pp38 and pJNK in this network seems less important than that of pERK and pCREB.

c-FOS mainly receives signals from pERK and pCREB. The effect of the 2nd order Volterra kernels *k*_2_ of pERK and pCREB is concentrated in the diagonal band, which corresponds to a time period of less than 50 minutes. This result allows us to infer that c-FOS reveals a positive synergistic effect following treatment with two impulsive inputs with a lag-time of 50 minutes between the two impulsive inputs (the region inside the dashed lines in Fig 3). This might reflect the priming effect from pERK to c-FOS reported previously [18]. To better illustrate the nonlinear contribution to the system, for nonlinear LF-PLS and also a model ignoring nonlinear terms, we plotted the simulated and theoretical c-FOS time course in the presence of two pulses and impulses of pERK and pCREB (Fig 4B). The second pulse or impulse induced a large increase of c-FOS intensity in the nonlinear model, when the time-interval is short in particular. In addition to c-FOS, the 1st order Volterra kernels *k*_1_ of EGR1 and c-JUN were similar in their responses to pERK, pCREB and pJNK, whereas the 2nd order Volterra kernel *k*_2_ was stronger in EGR1. This result suggests the nonlinear synergy of pERK and pCREB on EGR1, which is similar to the priming effect of pERK on c-FOS induction [18]. c-FOS and EGR1 are known to respond better to intermittent ERK activation as compared with sustained ERK activation [19], which also agrees with the result obtained for the 2nd order Volterra kernels. Thus, 2nd order Volterra kernels appear to be able to reveal synergistic regulation of gene expression by a signal transduction pathway.

### Effects of inputs on the VIP score

The upstream dependency of downstream pathway attracts much interest in signaling study. From a point of view of input-output system, the upstream dependency can be considered as a selection problem of input variables. A major framework of variable selection is model selection by use with information criteria, such as AIC. However, a variable importance of projections (VIP) score [20] has been conventionally used as a measure of variable selection in PLS regression. We used the average of the VIP scores with respect to upstream molecule (see methods), which is calculated from regression coefficient of Laguerre filter, as an index of upstream dependency. The average of the VIP scores shown in Fig 4A indicates that pERK and pCREB are important for c-FOS induction, consistent with earlier findings [11, 21]. Each IEG appears to respond to each of the MAPKs and pCREB, although with differing levels of response to the different inputs. This framework, the comparison of the mean VIP scores from each inputs, provides the unbiased interpretation of upstream dependency from experimental data.

### Similarity between input loadings reflects decoding characteristics

The LF-PLS yields the input loadings for the input space (S5 Fig). The loading is proportional to the correlation between the score and the original variable, and can be interpreted as the contribution of the original variable to the latent variable, which is the principal component. The input loadings are obtained with respect to each IEG output. Thus, we consider that the similarity between the input loadings of each output IEG reflects the decoding characteristics from the inputs, and thus, we applied the hierarchical clustering to the input loadings (Fig 4C). Namely, we applied the hierarchical clustering to the data set of concatenated input loadings matrix ${\left[T_{\text{c-FOS}}^{T},T_{\text{EGR1}}^{T},T_{\text{c-JUN}}^{T},T_{\text{JUNB}}^{T},T_{\text{FOSB}}^{T}\right]}^{T}$, where **T**_IEG_ is a input loading matrix of IEG. The two input loadings of JUNB and FOSB are the most similar to one another and to c-FOS. This suggests that JUNB and FOSB are downstream of c-FOS [11, 22, 23]. The input loadings of EGR1 are similar to those of c-JUN. This is consistent with our previous finding that EGR1 and c-JUN use pERK as an input [11]. The conclusions, based on the model, that c-FOS uses pERK and pCREB as inputs and that c-FOS is upstream of JUNB and FOSB, are consistent with previous experimental observations [11, 21].

## Discussion

We constructed the LF-PLS model which can be trained unbiasedly from experimental time-series data and applied this framework to infer the IO relationship between MAPKs and CREB, and the IEGs. Our results suggest a priming effect of pERK and pCREB on c-FOS induction. Notably, this finding was predicted by the model without using specific knowledge of the mechanisms underlying c-FOS activation [18]. Indeed, an advantage of data-driven modeling is that the model can extract characteristics of system without the specific knowledge of biochemical networks, even though the IO relationship inferred from the Laguerre filter does not necessarily mean direct molecular interactions. Furthermore, in this study we used PLS regression to estimate the parameters of the Laguerre filter, and found that the LF-PLS reproduced the time series data better than the ordinary LF.

The MSE of LOO CV of LF-PLS was not smaller than that of the FIR-PLS for EGR1 and c-JUN, despite the higher expression ability of IO relationships of LF-PLS as compared with that of FIR-PLS. The larger MSE of LOO CV of LF-PLS may be caused by over-fitting. Since the LF-PLS has a larger number of parameters than the FIR-PLS, the LF-PLS might require a larger sample size than the FIR-PLS to tune the parameters well. Thus, we consider that if the number of samples is sufficient, the performance of LF-PLS is either the same or better than that of the FIR-PLS. Furthermore, combining regularization methods such as *l*_2_ norm minimization [24] with PLS regression might reduce over-fitting of the LF-PLS. Other high-throughput technique, such as live-cell imaging using signaling reporters, now emerges to produce a lot more data points, which would be beneficial to apply our framework in the future.

We found a priming effect of pERK and pCREB on c-FOS induction by applying LF-PLS. Previous work [18] has shown c-FOS is expressed about 30 min after stimulation and degraded, with a half-life of approximately 30 min; however, its half-life is extended to more than 2 h when it is phosphorylated by ERK [25–27]. Our analysis of the 2nd order Volterra kernel predicted two pulses of pERK, with time lags of 50 min or less between them, will induce c-FOS strongly. This corresponds well to experimentally observed time series of c-FOS induction and degradation. However, although we inferred the findings from the second kernel correspond to this protein half-life regulation, it also could be other mechanisms such that involved in transcription factor recruitment and chromatin opening. The mechanism underlying the priming effect remains unknown and, further study is required to address the issue.

We previously used a nonlinear autoregressive exogenous model (nonlinear ARX model) for analysis of the same signaling network, instead of using the Laguerre filter [11]. A nonlinear ARX model consists of a combination of a linear ARX model and nonlinear transformation of the input using the Hill equation. The linear ARX model is a technique of system identification used in control engineering. Roughly speaking, a linear ARX model predicts the future time series of outputs based on the linear sum of a past time series of inputs and outputs. Although the nonlinear ARX model reproduced a time series for IEGs, interpretation of the response function of the nonlinear ARX model is not easy because the nonlinearity of ARX is heuristically introduced by the nonlinear transformation of input. By contrast, the nonlinearity of the Laguerre filter is introduced by the Volterra expansion; thus, the response function of the Laguerre filter is interpreted as an extension of the impulse response. In addition, the nonlinear ARX model includes autoregressive components, whereas the Laguerre filter applied in this study does not include autoregressive components. In other words, the nonlinear ARX model reproduces the time series of IEGs by pMAPKs, pCREB and each IEG, but the Laguerre filter reproduces the time series of IEGs by pMAPKs and pCREB without each IEGs. Thus, the Laguerre filter approach appears to be more widely applicable than use of a nonlinear ARX model in cases in which monitoring the outputs is not easy.

Statistical model selection by use with information criteria such as Akaike information criterion (AIC) [28] is a straightforward approach to determine inputs. In our previous study [11], the input was determined by exploring the AIC of all candidates of the models, which includes all combinations of inputs. (Note that, the number of input variables is at most 4, thus, we can brute-force explore all combination of inputs for multi input and single output nonlinear ARX model.) By contrast, we used the VIP score to examine the importance of inputs in this study. The three IO relationships between pCREB and EGR1, c-JUN, and FOSB, respectively, appeared only by VIP score (S7 Fig). On the other hand, the IO relationship between pCREB and JUNB appeared only by AIC of nonlinear ARX model. Thus, the results obtained using the VIP score analysis seems to show more redundancy in input selections as compared to what was predicted using the AIC approach.

In the previous study [11], c-FOS were selected because prior knowledge suggested they were candidate inputs to JUNB and FOSB [22]. In this study, c-FOS was not used as an input to JUNB and FOSB because we avoided applying prior knowledge. This may lead to a difference in c-FOS-dependency on JUNB and FOSB induction inferred using our previous approach versus with the current study. However, the VIP score of pCREB was the largest for both JUNB and FOSB, although the VIP score of pCREB for JUNB is smaller than 0.6. Given that c-FOS is downstream of pCREB [29], this result is reasonably consistent with the results of our previous study using AIC, in which c-FOS was selected as an input to JUNB and FOSB [11].

For input selection, the AIC is evaluated by eliminating inputs that are assumed not to affect outputs from the model. However, the VIP score is evaluated based on the full model, containing all inputs. Thus, AIC is more strict regarding input selection as compared to the VIP score approach. We reason that this might be one of the reasons the VIP score tends to be redundant. Furthermore, we propose that the VIP score is useful in the conventional point of view, as the VIP score has a lower computational cost than input selection by AIC. Together with the VIP score, the similarity between input loadings might help in selecting candidates of the model to further explore by AIC.

Reproducing strong nonlinearity is difficult to do using a Laguerre filter approach since higher Volterra kernels are required. Using a higher Volterra kernel increases the number of parameters, possibly leading to over-fitting and a huge computational cost for training of the model. However, empirical evidence suggests that strong nonlinearity is rare in signal transduction networks. Thus, the LF-PLS can be useful for analysis of many signal transduction pathways.

In conclusion, using data-driven modeling, we found a priming effect of c-FOS through the response function. This indicates that data-driven modeling is useful and identification of the IO relationships can help elucidate a biological mechanism. In addition, tackling the nonlinearity of biological systems remains a big challenge. The Laguerre filter can provide a relatively simple representation of the nonlinearity of living systems, and yields an interpretable response function, which can be regarded as a natural extension of the impulse response function. Parameter estimation of the nonlinear model is often difficult due to the high expression ability of model. However, we found that applying a Laguerre filter approach combined with PLS regression works effectively. Thus, we propose that the LF-PLS approach is widely applicable to a number of biological systems.

## Materials

### Antibodies

Mouse anti-phospho-ERK1/2 (Thr 202/Tyr 204) monoclonal antibody (mAb) (#9106), rabbit anti-phospho-CREB (Ser 133) mAb (#9198), rabbit anti-phospho-JNK (Thr183/Tyr185) mAb (#4668), rabbit anti-EGR1 mAb (#4154), rabbit anti-c-JUN mAb (#9165), rabbit anti-c-FOS mAb (#2250), rabbit anti-JUNB mAb (#3753) and rabbit anti-FOSB mAb (#2251) were purchased from Cell Signaling Technology (Beverly, MA). Rabbit anti-phospho-p38 mAb (v1211) was purchased from Promega (Madison, WI).

### Cell culture and treatments

PC12 cells (kindly provided by Masato Nakafuku, Cincinnati Children’s Hospital Medical Center, Ohio) [8] were cultured at 37°C under 5% CO_2_ in Dulbecco’s modified Eagle’s medium (DMEM) supplemented with 10% fetal bovine serum and 5% horse serum (Invitrogen, Carlsbad, CA), and stimulated by recombinant mouse b-NGF (R&D Systems, Minneapolis, MN), EGF (Roche, Mannheim, Germany), PACAP (Sigma, Zwijndrecht, The Netherlands), or anisomycin (EMD Biosciences, Inc., San Diego, CA) as previously described [8]. We used a low dose of anisomycin (50 nM) to activate p38 and JNK without inhibiting translation. For the inhibitor experiment, we stimulated cells with NGF in the presence of 10 nM PD (PD0325901, a MEK inhibitor, Sigma Zwijndrecht, The Netherlands), 5 mM H89 (PKA inhibitor, Sigma Zwijndrecht, The Netherlands). The inhibitors were added 30 min before growth factor stimulation. For the QIC assays, cells were seeded at a density of 104 cells per well in 96-well poly-L-lysine-coated glass-bottomed plates (Thermo Fisher Scientific, Pittsburgh, PA), and then starved in DMEM containing 25 mM HEPES and 0.1% bovine serum albumin for approximately 18 h before stimulation. Stimulations for cells seeded in 96-well microplates were performed by replacing the starvation medium with a medium containing the stimulant, using a liquid handling system (BiomekH NX Span-8, Beckman Coulter, Fullerton, CA) with an integrated heater-shaker (VariomagH, Daytona Beach, FL) and robotic incubator (STX-40, Liconic, Mauren, Liechtenstein). Note that all the cells within a plate were fixed simultaneously to prevent the exposure of the cells to formaldehyde vapor during the treatment.

### Quantitative Image Cytometry (QIC)

QIC [16] was performed as previously described. Briefly, after stimulation with growth factors, the cells were fixed, washed with phosphate-buffered saline (PBS), and permeabilised with blocking buffer (0.1% Triton X-100, 10% fetal bovine serum in PBS). The cells were washed and then incubated for 2 h with primary antibodies diluted in Can Get Signal immunostain Solution A (Toyobo, Osaka, Japan). The cells were washed three times and then incubated for 1 h with second antibodies. After immunostaining, the cells were stained with a marker of the nucleus (Hoechst 33342; Invitrogen) and of the cytoplasm (CellMask Deep Red stain; Invitrogen). Images of the stained cells were acquired using a CellWoRx (Thermo Fisher Scientific) automated microscope with an a610 objective. For QIC analyses, we acquired two different fields per well, making it possible to obtain data for an average of 1,238 +/- 356 cells per well (mean +/- 6 SD). All liquid handling for the 96-well microplates was performed using a BiomekH NX Span-8 liquid handling system. Intensities of the signaling activity and the IEGs between experiments were normalized to an internal control included on each 96-well plate.

## Methods

### Laguerre filter

A nonlinear single input single output (nonlinear SISO) system with the input *u*(*t*) and the output *y*(*t*) at time *t*

y(t)=F[t,u(t))], (3)

can be expanded using a Volterra series [12]. We assume that the system is approximated by the second order Volterra model

$$y(t)\approx k_{0}(t)+\int d\tau_{1}k_{1}(\tau_{1})u(t-\tau_{1})+\int d\tau_{1}d\tau_{2}k_{2}(\tau_{1},\tau_{2})u(t-\tau_{1})u(t-\tau_{2}),$$ (4)

where *k*_0_,*k*_1_ and *k*_2_ are the zero-th order, the first order and the second order Volterra kernel, respectively. Hereafter, we set *k*_0_ to a constant value.

Similarly, a nonlinear multiple input single output (nonlinear MISO) system model with *m* input *u*^(*i*)^(*t*),*i* = 1,⋯,*m* and output *y*(*t*)

$$y(t)=F[t,u^{\left(1\right)}(t),\cdots ,u^{\left(m\right)}(t)],$$ (5)

is approximated by the second order Volterra model

$$y(t)\approx k_{0}+\sum_{i=1}^{m}\int d\tau_{1}k_{1}^{\left(i\right)}(\tau_{1})u^{\left(i\right)}(t-\tau_{1})+\sum_{i,j=1}^{m}\int d\tau_{1}d\tau_{2}k_{2}^{\left(i,j\right)}(\tau_{1},\tau_{2})u^{\left(i\right)}(t-\tau_{1})u^{\left(j\right)}(t-\tau_{2}).$$ (6)

The kernel is expanded by the Laguerre basis function, which is one of the orthogonal basis functions

$$k_{1}^{\left(i\right)}(\tau_{1})=\sum_{a=1}^{\infty }c_{a}^{\left(i\right)}l_{a}(\tau_{1}),k_{2}^{\left(i,j\right)}(\tau_{1},\tau_{2})=\sum_{a,b=1}^{\infty }d_{a,b}^{\left(i,j\right)}l_{a}(\tau_{1})l_{b}(\tau_{2}),$$ (7)

where *l*_*n*_(*t*) is *n*-th order Laguerre function defined by

$$l_{n}\left(t\right)=\sqrt{2p}e^{pt}\frac{1}{\left(n-1\right)!}{\left(\frac{d}{dt}\right)}^{n-1}t^{n-1}e^{-2pt}.$$ (8)

Substituting Eq (7) into Eq (6),

$$y(t)\approx k_{0}+\sum_{i=1}^{m}\sum_{a=1}^{\infty }c_{a}^{\left(i\right)}x_{a}^{\left(i\right)}(t)+\sum_{i,j=1}^{m}\sum_{a,b=1}^{\infty }d_{a,b}^{\left(i,j\right)}x_{a}^{\left(i\right)}(t)x_{b}^{\left(j\right)}(t),$$ (9)

where $x_{a}^{\left(i\right)}(t)\equiv \int d\tau l_{a}(\tau )u^{\left(i\right)}(t-\tau )$. We truncated the order of the Laguerre basis function at a finite positive integer at *m*_*l*_ and ignored the cross term of the second Volterra kernel for simplicity,

$$y(t)\approx k_{0}+\sum_{i=1}^{m}\left\{\sum_{a=1}^{m_{l}}c_{a}^{\left(i\right)}x_{a}^{\left(i\right)}(t)+\sum_{a,b=1}^{m_{l}}d_{a,b}^{\left(i\right)}x_{a}^{\left(i\right)}(t)x_{b}^{\left(i\right)}(t)\right\}.$$ (10)

This approximation means that the interaction between impulses of input *i* and *j* is ignored.

### Implementation of the Laguerre filter

The vector $x^{\left(i\right)}(t)={\left(x_{1}^{\left(i\right)}(t),\cdots ,x_{m}^{\left(i\right)}(t)\right)}^{T}$ satisfies the ordinal differential equation

$$\frac{dx^{\left(i\right)}(t)}{dt}=Ax^{\left(i\right)}(t)+\sqrt{2p}\left(\begin{array}{c} 1\\ \vdots \\ 1 \end{array}\right)u(t),$$ (11)

$$A\equiv \left(\begin{array}{l} \begin{array}{cc} -p & \begin{array}{cc} \;0 & \begin{array}{cc} \cdots & \begin{array}{cc} \cdots & 0 \end{array} \end{array} \end{array} \end{array}\\ \begin{array}{cc} -2p & \begin{array}{cc} -p & \begin{array}{cc} 0 & \begin{array}{cc} \cdots & 0 \end{array} \end{array} \end{array} \end{array}\\ \begin{array}{cc} -2p & \begin{array}{cc} \cdots & \begin{array}{cc} \ddots & \begin{array}{cc} \cdots & 0 \end{array} \end{array} \end{array} \end{array}\\ \begin{array}{cc} \;\vdots & \begin{array}{cc} \; & \begin{array}{cc} & \begin{array}{cc} \ddots & \vdots \end{array} \end{array} \end{array} \end{array}\\ \begin{array}{cc} -2p & \begin{array}{cc} \cdots & \begin{array}{cc} -2p & -p \end{array} \end{array} \end{array} \end{array}\right),$$ (12)

where *p* is a scale parameter of the Laguerre basis function [30]. Thus, we obtain the value of **X**^(*i*)^(*t*) by solving Eq (11).

### Parameter estimation by partial least square regression

The parameters $\left\{c_{a}^{\left(i\right)}\},\{d_{a,b}^{\left(i\right)}\right\}$ are estimated using the partial least square (PLS) regression method [13]. The Eq (10) can be rewritten as **y** = **Xβ**+**ε** where $y={\left(y(t_{1}),\cdots ,y(t_{n})\right)}^{T},$x1:ml(1:m)(t)=(x1(1),⋯,x,ml(1)⋯,x1(m),⋯,xml(m))T,

$$X=\left(\begin{array}{l} \begin{array}{cc} \begin{array}{cc} 1 & x_{1:m_{l}}^{\left(1:m\right)}{\left(t_{1}\right)}^{T} \end{array} & \text{vec}{\left({\left\{x_{1:m_{l}}^{\left(1:m\right)}(t_{1})x_{1:m_{l}}^{\left(1:m\right)}{\left(t_{1}\right)}^{T}\right\}}_{i\geq j}\right)}^{T} \end{array}\\ \begin{array}{cc} \begin{array}{cc} \cdots & \cdots \end{array} & \cdots \end{array}\\ \begin{array}{cc} \begin{array}{cc} 1 & x_{1:m_{l}}^{\left(1:m\right)}{\left(t_{k}\right)}^{T} \end{array} & \text{vec}{\left({\left\{x_{1:m_{l}}^{\left(1:m\right)}(t_{k})x_{1:m_{l}}^{\left(1:m\right)}{\left(t_{k}\right)}^{T}\right\}}_{i\geq j}\right)}^{T} \end{array} \end{array}\right),$$

vec(**A**) ≡ (*a*_11_,⋯,*a*_ij_,⋯)^*T*^, **β** is a regression coefficient parameter.

This formulation can easily be applied to multiple samples of input and outputs, ${\left\{X_{r},y_{r}\right\}}_{r=1}^{n}$ by redefining ***X*** and ***y*** as $\tilde{X}={\left(X_{1}^{T},\cdots ,X_{n}^{T}\right)}^{T}$,$\tilde{y}={\left(y_{1}^{T},\cdots ,y_{n}^{T}\right)}^{T}$, where

$$X_{r}=\left(\begin{array}{l} \begin{array}{cc} \begin{array}{cc} 1 & x_{1:m_{l},r}^{\left(1:m\right)}{\left(t_{1}\right)}^{T} \end{array} & \text{vec}{\left({\left\{x_{1:m_{l},r}^{\left(1:m\right)}(t_{1})x_{1:m_{l},r}^{\left(1:m\right)}{\left(t_{1}\right)}^{T}\right\}}_{i\geq j}\right)}^{T} \end{array}\\ \begin{array}{cc} \begin{array}{cc} \cdots & \cdots \end{array} & \cdots \end{array}\\ \begin{array}{cc} \begin{array}{cc} 1 & x_{1:m_{l},r}^{\left(1:m\right)}{\left(t_{k}\right)}^{T} \end{array} & \text{vec}{\left({\left\{x_{1:m_{l},r}^{\left(1:m\right)}(t_{k})x_{1:m_{l},r}^{\left(1:m\right)}{\left(t_{k}\right)}^{T}\right\}}_{i\geq j}\right)}^{T} \end{array} \end{array}\right),$$

***y***_r_ = (*y*_r_(*t*_1_),⋯,*y*_r_(*t*_*n*_))^*T*^, *r* represents the sample number for the time series. We determined the regression coefficient parameter **β** using the PLS regression method.

### Determining the number of PLS components

Experimental data for all 7 conditions were used to implement the Laguerre filter in order to determine the number of PLS components. Laguerre basis functions up to the 9th order, including the 0th order (*m*_*l*_ = 10 in the Eq (10)), were used in this study, with the assumption that 10 Laguerre basis functions would be sufficient to represent the system. The scale parameter *p* and the regression coefficient β were estimated by alternately minimizing the training MSE until it had converged. To avoid over-fitting, the initial value of the scale parameter *p* in Eq (11) was estimated using only the 0th and 1st order Laguerre basis functions. As the number of PLS components increases, the percent increase of variance explained in outputs generally gets smaller. We determined the number of components such that the percent increase of variance falls below 2% with the addition of the last component.

### PLS regression model

In case of single output *y* and *m* input variables, PLS regression model with *k* latent variables $\left(k\leq \tilde{m}\right)$ can be described as follows

$$\begin{array}{l} X=TP^{T}+E\\ y=Tb+\varepsilon , \end{array}$$ (13)

where *X*,*T*,*P*,*y*,*b* are data matrix, input score matrix, input loading matrix, output vector and regression coefficient of input score matrix, respectively. *E*,*ε* are residual matrix and vector, respectively.

### VIP score

The VIP score for *j*-th variable is defined as

$${\text{VIP}}_{j}=\sqrt{\frac{\tilde{m}\sum_{k=1}^{h}b_{k}t_{k}^{T}t_{k}{\left(\frac{w_{jk}}{\left\|w_{k}\right\|}\right)}^{2}}{\sum_{k=1}^{h}b_{k}t_{k}^{T}t_{k}}},$$ (14)

where $\tilde{m}$, *t*_*k*_, *b*_*k*_ are the number of variables, *k*-th input score vector, regression coefficients of input score matrix **T**, respectively. *w*_*jk*_ is the PLS weight of the *k*-th variable for the *j*-th latent variable.

### The network modules and input patterns for artificial data production

The seven network modules and ten input patterns were used. Most of the parameters used for the network modules and input patterns are adapted from previous work [15], with a few alterations. 0.5 was used for a steady state value for input pattern 4, and the logistic function

$$F_{7}\left(t\right)=\frac{1}{1+e^{-0.05\left(t-150\right)}},$$ (15)

was used for input pattern 7. Each modules was simulated using the input patterns to produce 301 output time points. Then each models was trained using input patterns 1–8 and tested against input patterns 9 and 10 in order to calculate MSE.

### Computational and theoretical pulse and impulse responses

For the computational simulation, a triangular pulse of either pERK or pCREB with the maximum amplitude 0.5 were used as input. This pulse was given at time 0 and at 18 min or 90 min. For the model without nonlinearity, the corresponding parts of the regression coefficient parameters β were set to zero. For the impulse responses, output is calculated by

$$y\left(t\right)=Ak_{1}\left(t\right)+Bk_{1}\left(t-\tau \right)+A^{2}k_{2}\left(t,t\right)+2ABk_{2}\left(t,t-\tau \right)+B^{2}k_{2}\left(t-\tau ,t-\tau \right),$$ (16)

where *A* indicates an amplitude of an impulse at time 0 and *B* indicates an amplitude of an impulse at time *τ*. In our case, *A* is set to 0.5 and *B* is ranged from 0 to 1. For the model without nonlinearity, any terms having *k*_2_ are ignored.

## Supporting Information

S1 Fig pMAPKs and pCREB as inputs, and immediate early gene expression as outputs. (A) Time series of responses of pMAPKs and pCREB (circles) to NGF (0.5 ng/ml, red), PACAP (1 nM, blue), or EGF (0.5 ng/ml, green). Responses were measured by QIC at 3-min intervals over a total period of 180 min. (B) Time series showing the expression of immediate early genes (IEGs) (circles), together with results of a simulation of the Laguerre filter combined with PLS regression (solid lines). The color code is that same as that used in panel A.
(TIF)

S2 Fig Boxplots of Residuals of LOO CVs. (A) For each IEGs, a boxplot of residual distribution against each round of LOO CV dataset over seven conditions is shown in a panel. A red line, blue box and whisker indicate the median, the interquartile range (IQR), the end point of data point, which is not outlier. A red marker + indicates the outlier. A data point, which is smaller than Q1-1.5*IQR, or larger than Q3+1.5*IQR is detected as outlier, where Q1 and Q3 are 1st quartile and 3rd quartile, respectively. (B) The full scale box plot of ordinary LF is shown.
(TIF)

S3 Fig Boxplots of coefficient parameters of LOO CV and parameters of the final LF-PLS model. For each regression coefficients, distribution over LOO CVs are plotted as a box plot. The parameters from the final model that were trained with all the datasets are shown as green circles.
(TIF)

S4 Fig The boxplot of squared residuals of LOO CV. (A) For each IEGs, the squared residual of LF-PLS and FIR-PLS against LOC CVs over seven conditions are shown by boxplot. (B) For each IEGs, the logarithm of squared residual of each model against LOC CVs over seven conditions is shown by boxplot.
(TIF)

S5 Fig Loadings and scores for the inputs and outputs. In all panels, the vertical axis is the 1st principal component (PC1) and the horizontal axis is the 2nd principal component (PC2). For the panels showing input and output scores, a red circle indicates 5ng/ml NGF; red diamond, 0.5 ng/ml NGF; blue circle, 100 nM PACAP; blue diamond, 1 nM PACAP; green circle, 5 ng/ml EGF; green diamond, 0.5 ng/ml EGF; and black circle, 50 ng/ml Anisomycin. The color gradation of each marker changed from low to high over time. For the panels showing input and output loadings, blue indicates pERK; green, pCREB; red, pJNK; and cyan, pp38. Circle, 1st Volterra kernel; diamond, 2nd Volterra kernel.
(TIF)

S6 Fig The mean square error of training data set and leave one out error of cross validation of the four models. Blue, c-FOS; cyan, EGR1; green, c-JUN; orange, JUNB; red, FOSB. Ordinary FIR, a finite impulse response model combined with ordinary regression; FIR-PLS, a finite impulse response model combined with partial least square (PLS) regression; ordinary LF, an ordinary Laguerre filter combined with ordinary regression; LF-PLS, a Laguerre filter combined with PLS regression.
(TIF)

S7 Fig The IO relationships between signaling molecules and IEGs estimated by VIP score. Black solid line, gray solid line, dashed line are the IO relationship estimated by both VIP score and AIC of nonlinear ARX model, only VIP score, and only AIC of nonlinear ARX model, respectively. Dotted line is IO relationship estimated by AIC of nonlinear ARX model. c-FOS is used as input for output of JUNB and FOSB only in nonlinear ARX model. We regarded that the IO relationship exists if the average VIP score is more than 0.6.
(TIF)

S1 Table The mean square error (MSE) of the training data set. (TIF)

S2 Table The mean square error (MSE) of leave one out error of cross validation (LOO CV). (TIF)

S3 Table The p-values of the Mann—Whitney U-test for squared residuals between LF-PLS and FIR-PLS are shown. (TIF)
