# Supplementary figures and images for: Laguerre Filter Analysis with Partial Least Square Regression Reveals a Priming Effect of ERK and CREB on c-FOS Induction
Source: PLoS One. 2016 Aug 11;11(8):e0160548. doi: 10.1371/journal.pone.0160548 (PMC4981404; doi:10.1371/journal.pone.0160548)

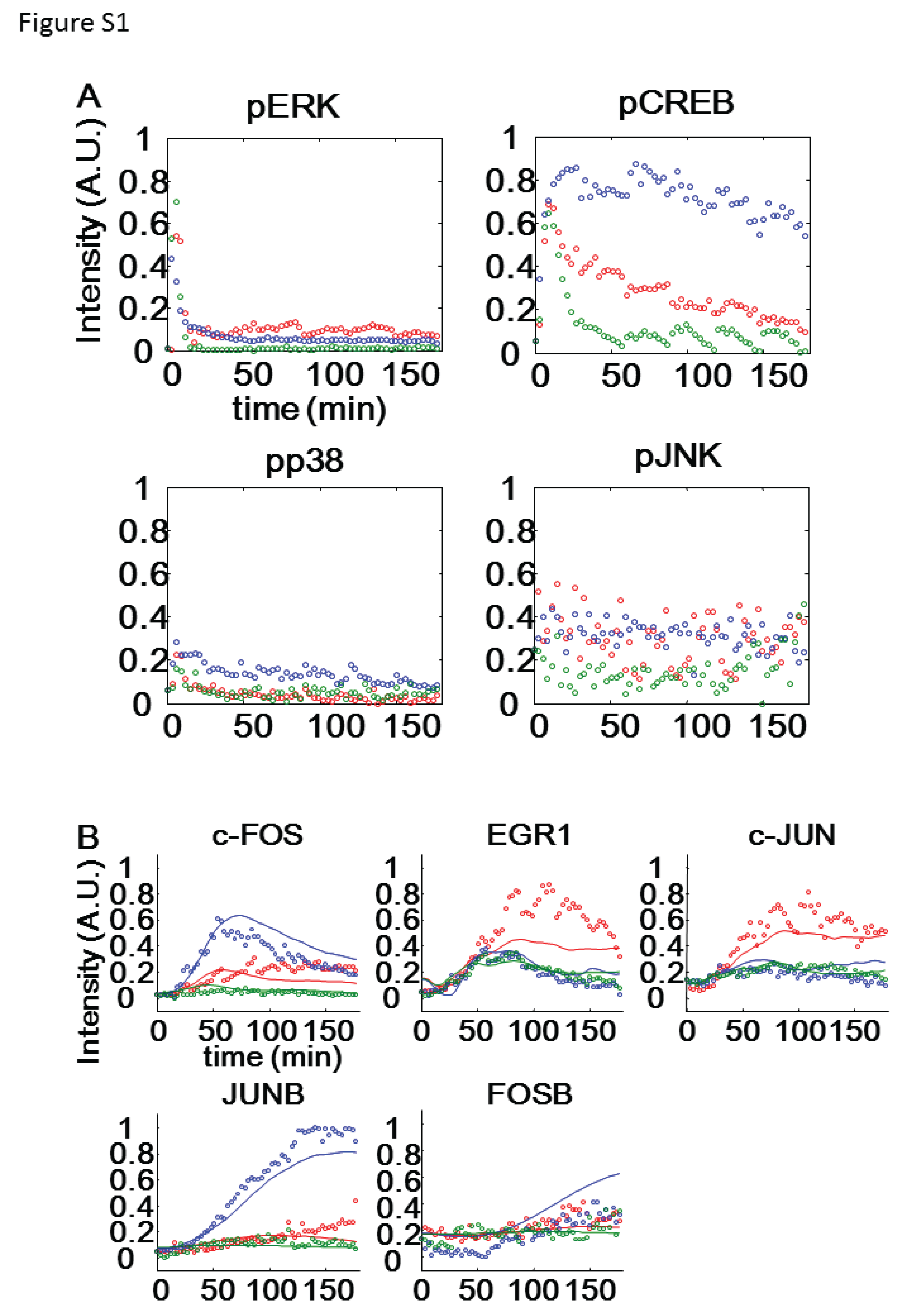

Supplement: S1 Fig — (A) Time series of responses of pMAPKs and pCREB (circles) to NGF (0.5 ng/ml, red), PACAP (1 nM, blue), or EGF (0.5 ng/ml, green). Responses were measured by QIC at 3-min intervals over a total period of 180 min. (B) Time series showing the expression of immediate early genes (IEGs) (circles), together with results of a simulation of the Laguerre filter combined with PLS regression (solid lines). The color code is that same as that used in panel A. (TIF) [file pone.0160548.s001.tif]

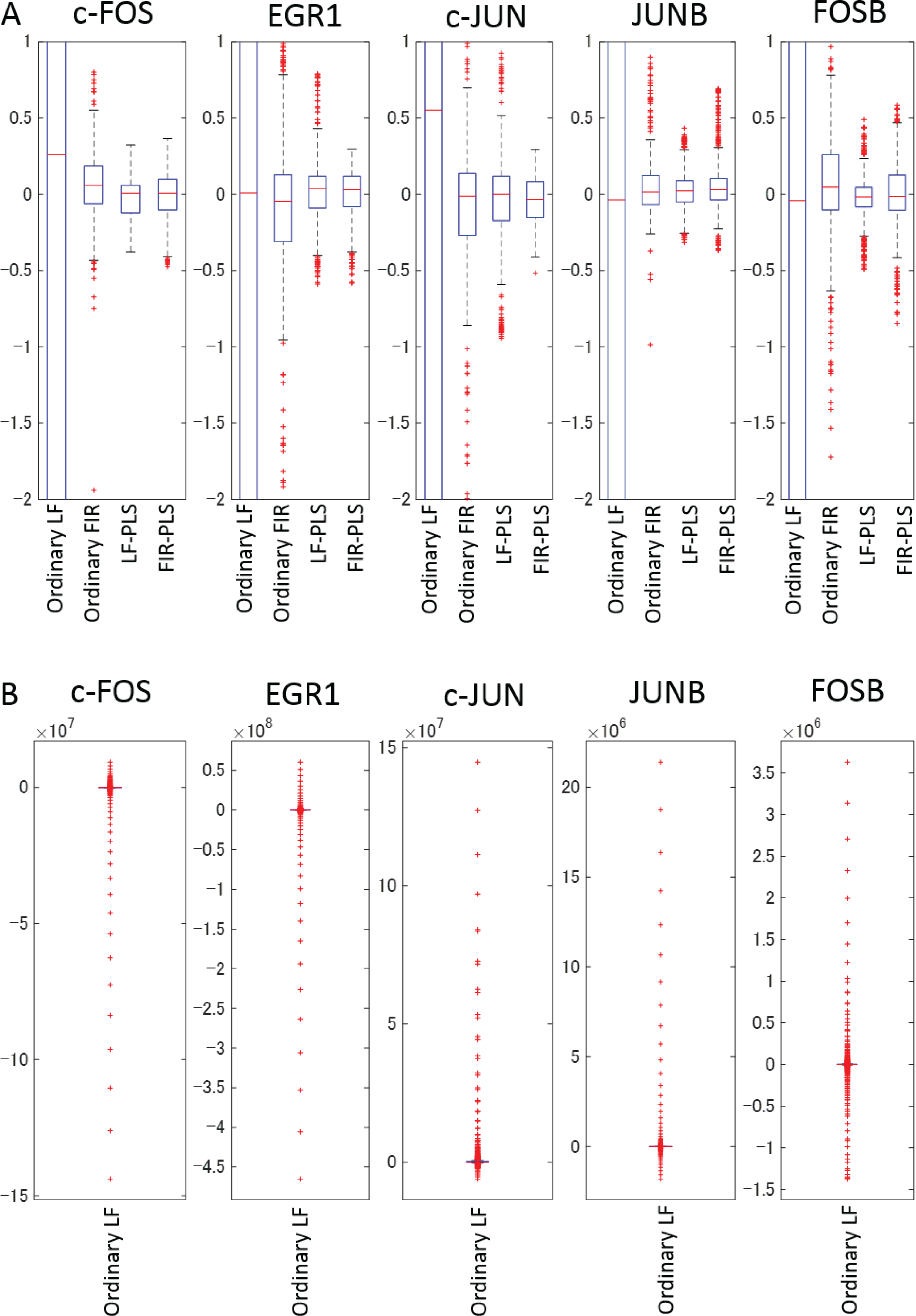

Supplement: S2 Fig — (A) For each IEGs, a boxplot of residual distribution against each round of LOO CV dataset over seven conditions is shown in a panel. A red line, blue box and whisker indicate the median, the interquartile range (IQR), the end point of data point, which is not outlier. A red marker + indicates the outlier. A data point, which is smaller than Q1-1.5*IQR, or larger than Q3+1.5*IQR is detected as outlier, where Q1 and Q3 are 1st quartile and 3rd quartile, respectively. (B) The full scale box plot of ordinary LF is shown. (TIF) [file pone.0160548.s002.tif]

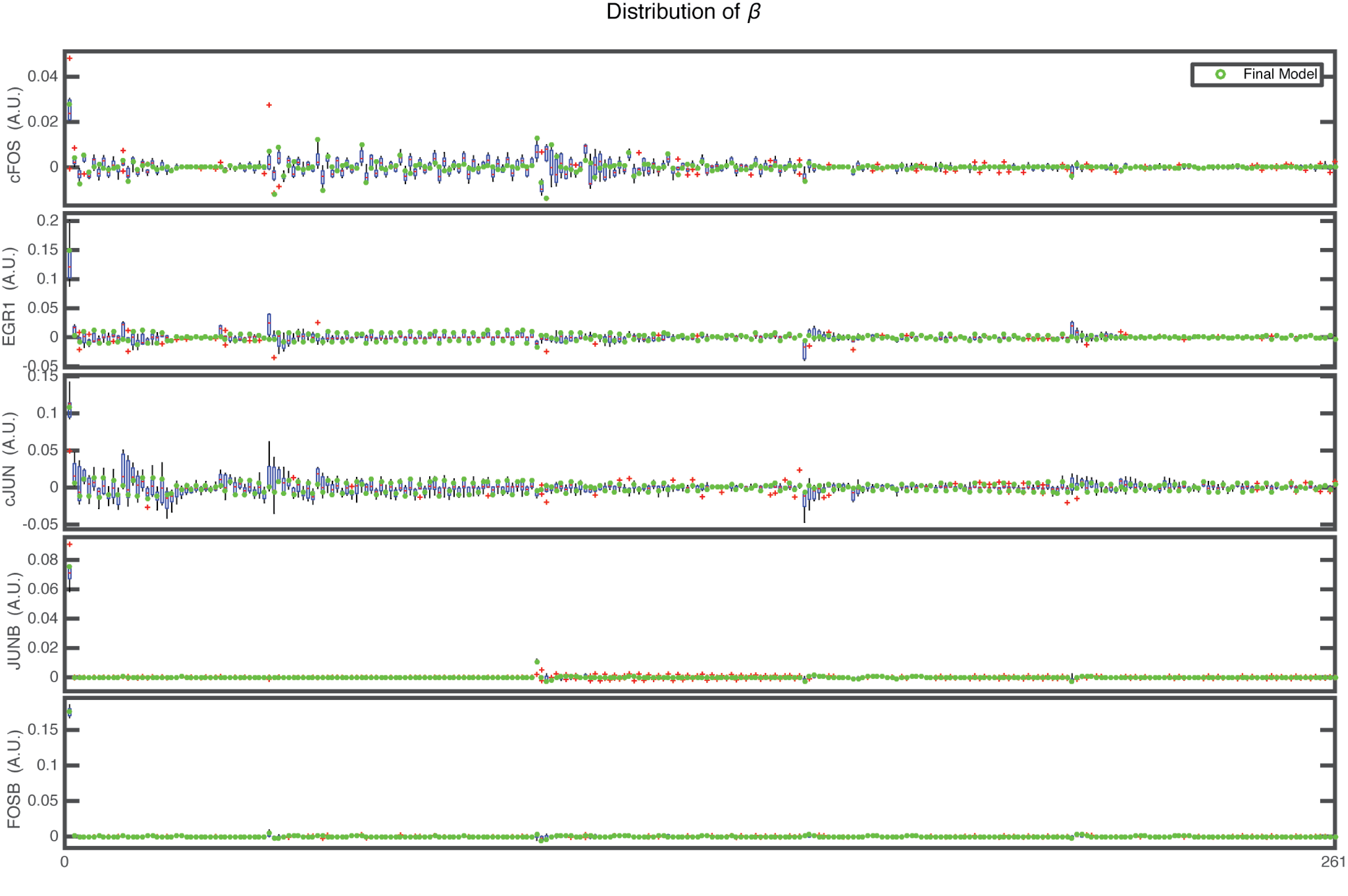

Supplement: S3 Fig — For each regression coefficients, distribution over LOO CVs are plotted as a box plot. The parameters from the final model that were trained with all the datasets are shown as green circles. (TIF) [file pone.0160548.s003.tif]

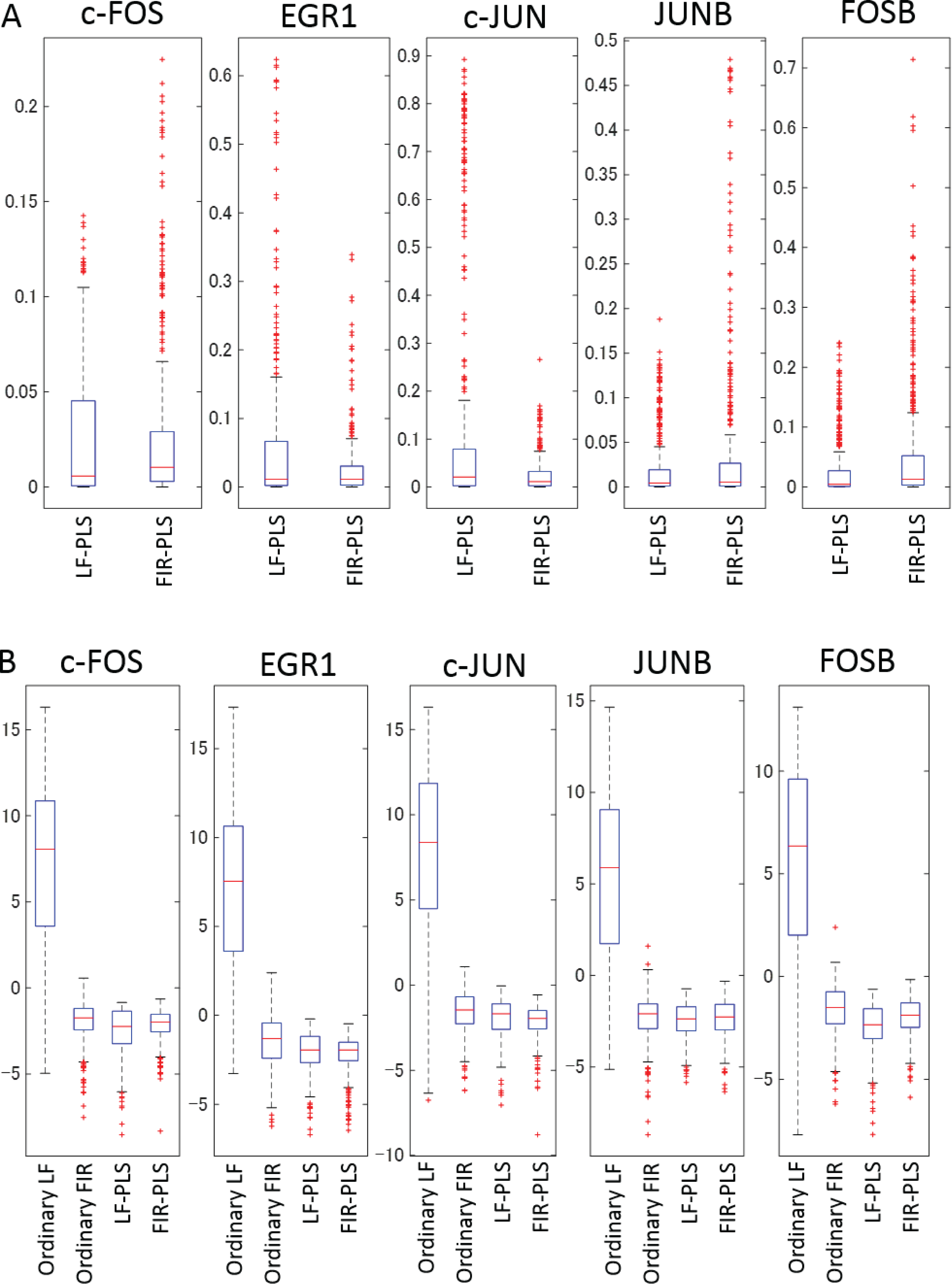

Supplement: S4 Fig — (A) For each IEGs, the squared residual of LF-PLS and FIR-PLS against LOC CVs over seven conditions are shown by boxplot. (B) For each IEGs, the logarithm of squared residual of each model against LOC CVs over seven conditions is shown by boxplot. (TIF) [file pone.0160548.s004.tif]

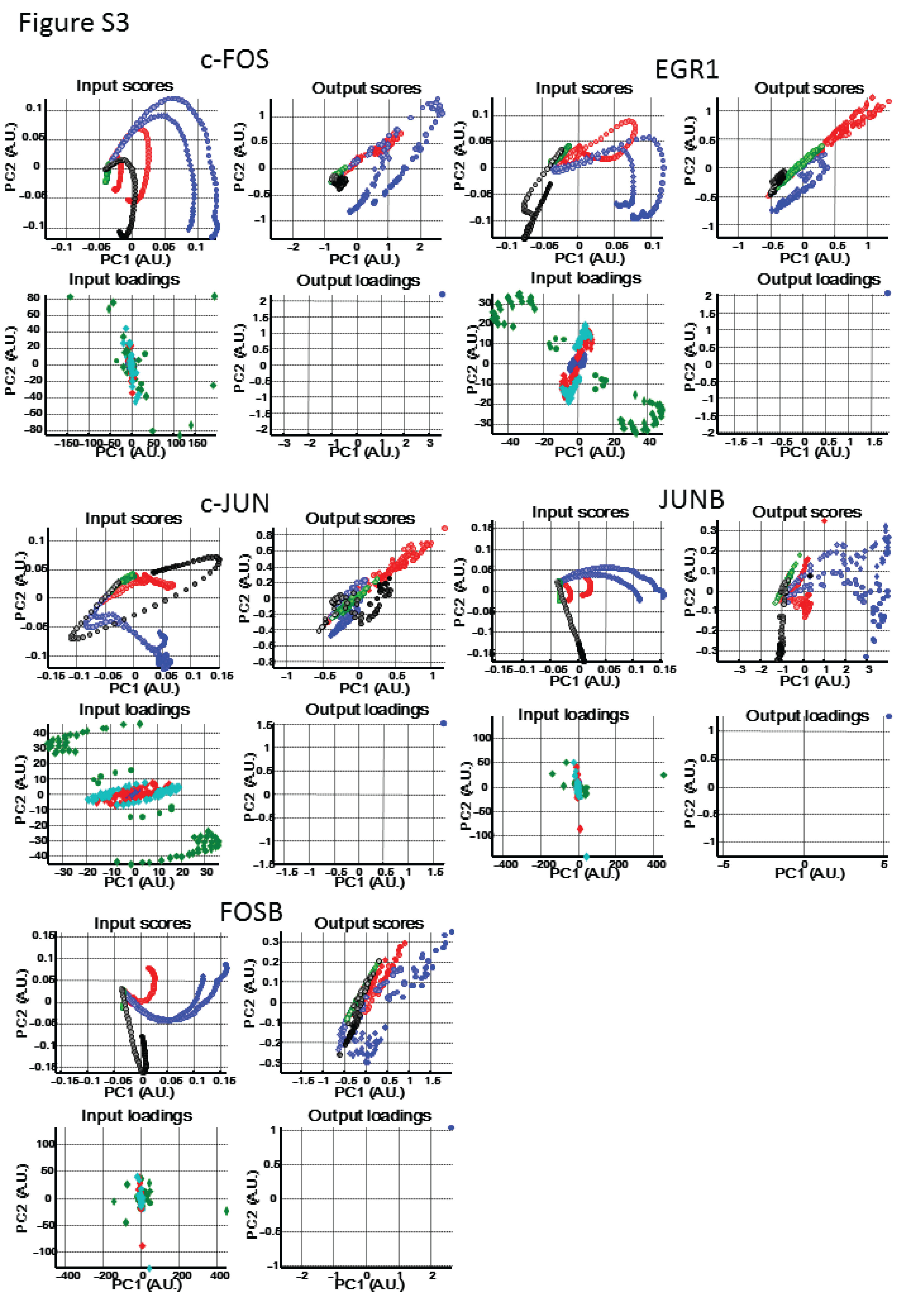

Supplement: S5 Fig — In all panels, the vertical axis is the 1st principal component (PC1) and the horizontal axis is the 2nd principal component (PC2). For the panels showing input and output scores, a red circle indicates 5ng/ml NGF; red diamond, 0.5 ng/ml NGF; blue circle, 100 nM PACAP; blue diamond, 1 nM PACAP; green circle, 5 ng/ml EGF; green diamond, 0.5 ng/ml EGF; and black circle, 50 ng/ml Anisomycin. The color gradation of each marker changed from low to high over time. For the panels showing input and output loadings, blue indicates pERK; green, pCREB; red, pJNK; and cyan, pp38. Circle, 1st Volterra kernel; diamond, 2nd Volterra kernel. (TIF) [file pone.0160548.s005.tif]

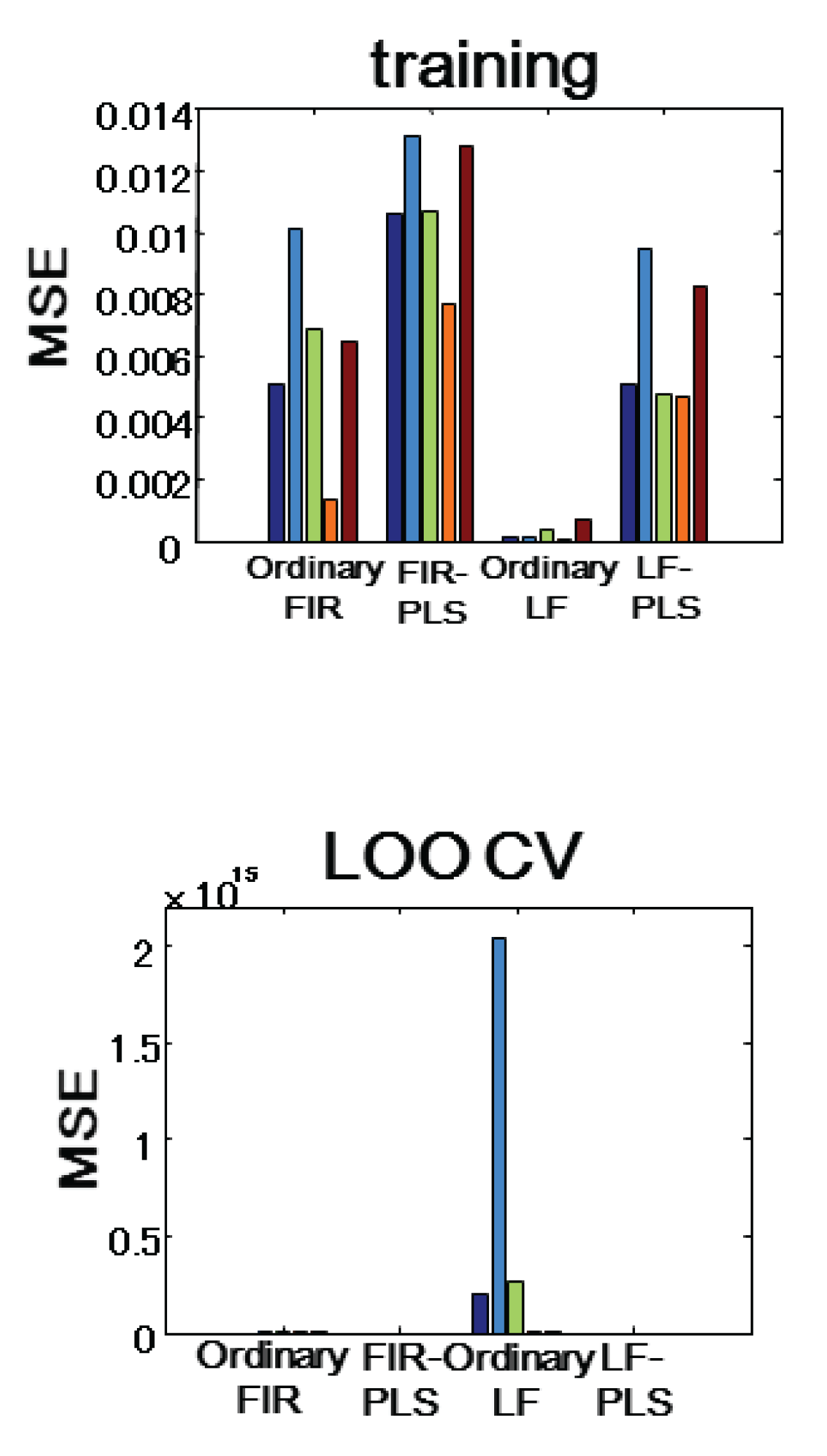

Supplement: S6 Fig — Blue, c-FOS; cyan, EGR1; green, c-JUN; orange, JUNB; red, FOSB. Ordinary FIR, a finite impulse response model combined with ordinary regression; FIR-PLS, a finite impulse response model combined with partial least square (PLS) regression; ordinary LF, an ordinary Laguerre filter combined with ordinary regression; LF-PLS, a Laguerre filter combined with PLS regression. (TIF) [file pone.0160548.s006.tif]

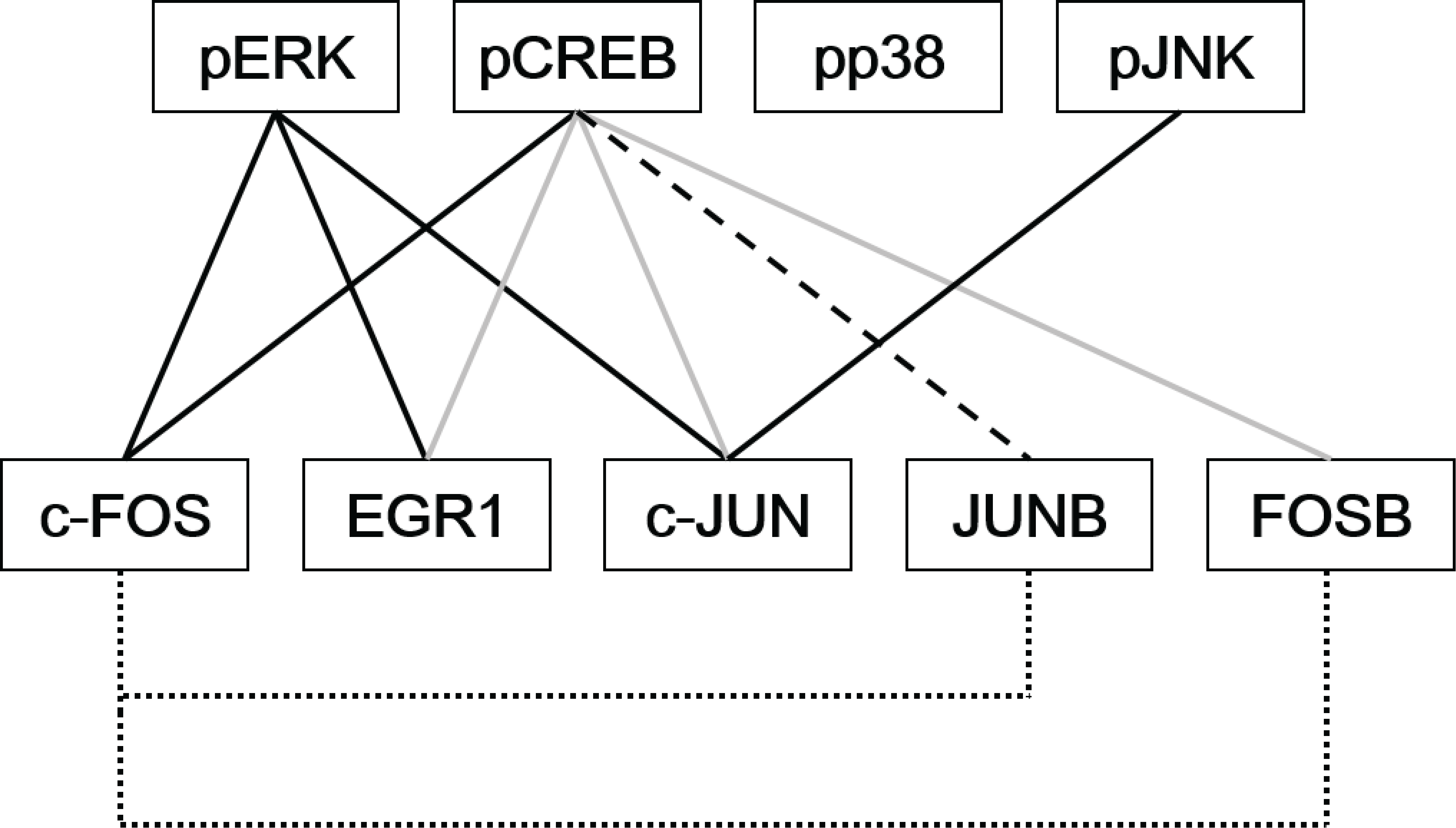

Supplement: S7 Fig — Black solid line, gray solid line, dashed line are the IO relationship estimated by both VIP score and AIC of nonlinear ARX model, only VIP score, and only AIC of nonlinear ARX model, respectively. Dotted line is IO relationship estimated by AIC of nonlinear ARX model. c-FOS is used as input for output of JUNB and FOSB only in nonlinear ARX model. We regarded that the IO relationship exists if the average VIP score is more than 0.6. (TIF) [file pone.0160548.s007.tif]

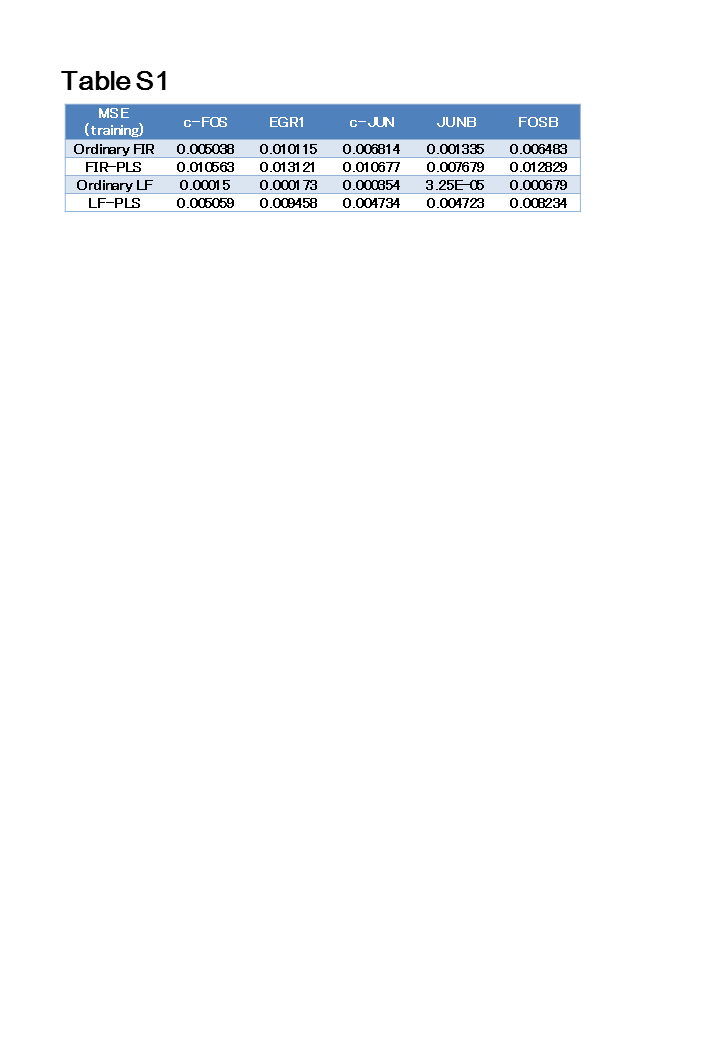

Supplement: S1 Table — (TIF) [file pone.0160548.s008.tif]

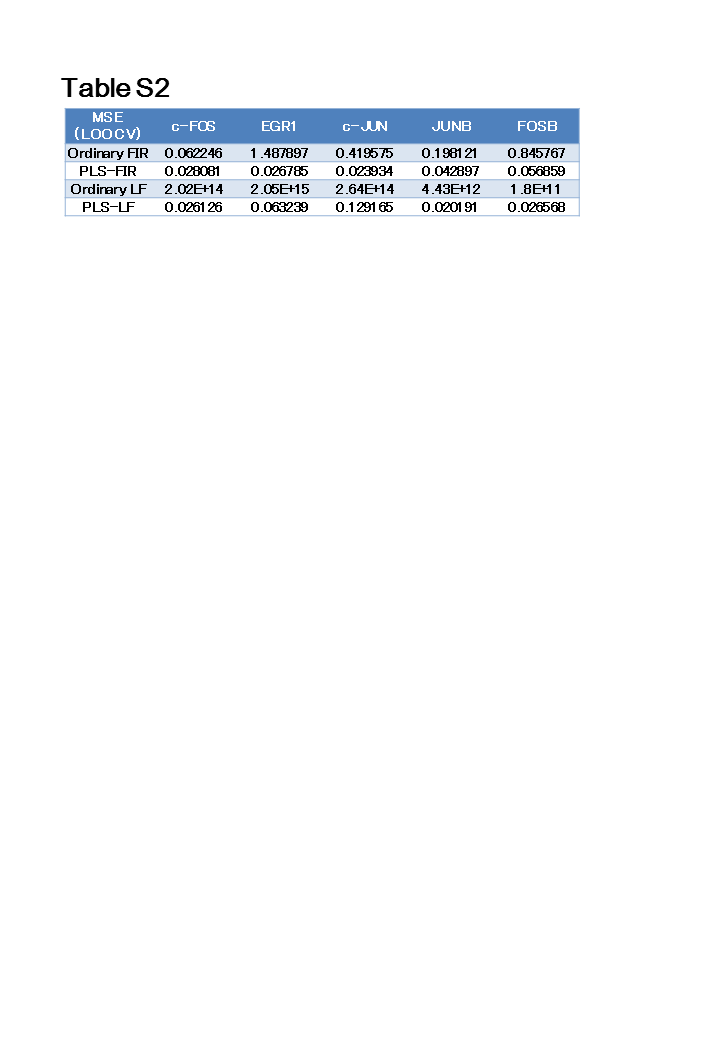

Supplement: S2 Table — (TIF) [file pone.0160548.s009.tif]

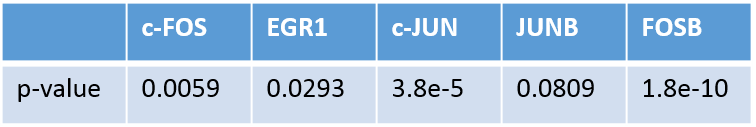

Supplement: S3 Table — (TIF) [file pone.0160548.s010.tif]
